# Impact of precursor solution temperature on two-step spin-coated FAPbI_3_ film elucidated by surface morphology and *in situ* photoluminescence dynamics

**DOI:** 10.1039/d5ra08868b

**Published:** 2026-01-22

**Authors:** Ryota Okuyama, Kohei Uezono, Yuhi Inada, Takeshi Yamao, Daichi Okada, Kenichi Yamashita

**Affiliations:** a Faculty of Electrical Engineering and Electronics, Kyoto Institute of Technology Matsugasaki, Sakyo-ku Kyoto 606-8585 Japan yamasita@kit.ac.jp; b Faculty of Materials Science and Engineering, Kyoto Institute of Technology Matsugasaki, Sakyo-ku Kyoto 606-8585 Japan

## Abstract

Perovskite solar cells fabricated by the two-step spin-coating method are highly sensitive to processing conditions, yet the role of precursor solution temperature remains poorly understood. Here, we systematically investigate how the temperatures of PbI_2_ and formamidinium iodide (FAI) precursor solutions affect crystallization dynamics, surface morphology, phase formation, and optoelectronic properties of FAPbI_3_ thin films. By combining microscopic characterization with *in situ* photoluminescence (PL) monitoring during annealing, we reveal distinct temperature-dependent nucleation, ripening, and film densification processes. PbI_2_ solutions at intermediate temperatures (50–70 °C) yield compact underlayers that promote homogeneous conversion and suppress residual PbI_2_, while low-temperature FAI solutions favor stabilization of the photoactive α-phase. Quantitative analysis of the *in situ* PL evolution clarifies the correlation between crystallization stages and optical out-coupling behavior. These results establish precursor solution temperature as a critical and practical parameter for controlling perovskite film formation in two-step deposition processes.

## Abstract 2

Precursor solution temperature governs crystallization, morphology, and phase formation in two-step spin-coated FAPbI_3_ films. *In situ* PL reveals nucleation behavior, showing that 50–70 °C for PbI_2_ and ∼25 °C for FAI yield compact α-phase films.

## Graphical abstract

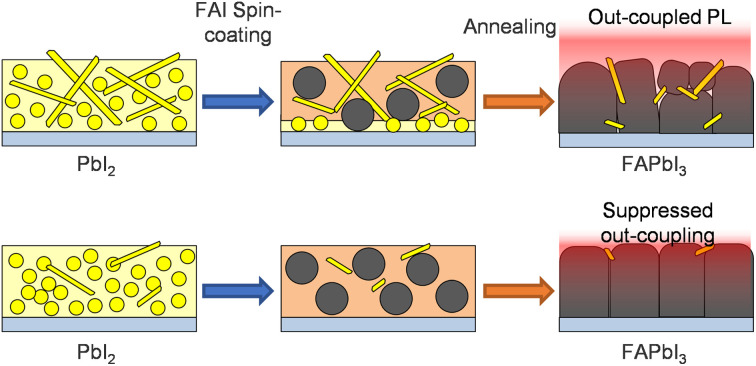

## 1 Introduction

Perovskite solar cells (PSCs) have attracted considerable attention as promising next-generation photovoltaic devices owing to their excellent optoelectronic properties,^1–3^ low-cost fabrication processes, and compatibility with flexible device architectures.^4^ In recent years, remarkable improvements in device performance have been achieved through advances in device architectures and material engineering, with single-junction PSCs now reporting power conversion efficiencies (PCEs) surpassing 26%,^5^ thereby accelerating research toward commercialization.^6–8^ Achieving high-efficiency PSCs requires the formation of high-quality perovskite thin films, for which the spin-coating method is the most widely employed film-deposition technique. Spin-coating approaches are generally classified into two categories, namely the one-step and two-step methods. In the one-step method, all precursors are mixed in a solution and deposited onto the substrate in a single spin-coating process.^9–11^ This approach offers the advantages of a simple procedure and ease of optimization. However, it often results in smaller crystal grain sizes and films with numerous pinholes and grain boundaries, which can lead to degradation in device performance.^12^

In contrast, the two-step spin-coating method involves sequential deposition of an inorganic precursor (typically PbI_2_) and an organic precursor (such as formamidinium iodide, FAI, and methylammonium iodide, MAI) in two separate spin-coating steps.^13–16^ For fabricating FAPbI_3_ films, PbI_2_ is first dissolved in a mixed solvent of *N*,*N*-dimethylformamide (DMF) and dimethyl sulfoxide (DMSO), spin-coated onto the substrate and then thermally annealed. Subsequently, an FAI solution in isopropanol (IPA) is spin-coated onto the PbI_2_ layer to form the perovskite film. Because the solubility of PbI_2_ in IPA is low, the underlying PbI_2_ layer is retained during the second step. During this process, interdiffusion between PbI_2_ and FAI occurs, leading to the formation of a dense and highly crystalline perovskite thin film. In addition to their extensive use in photovoltaic devices, FAPbI_3_ have also been explored in other optoelectronic material platforms, such as luminescent nanocrystals for sensing and lighting applications. For example, double-encapsulated red-emitting FA-based perovskite nanocrystals have been reported to exhibit enhanced optical stability and emission robustness.^17^ These studies underline the intrinsic versatility of FA-based perovskites and further motivate a detailed understanding of their crystallization behavior and phase formation under solution-processed conditions.

Extensive studies have investigated the two-step spin-coating method, addressing factors such as the ratio of organic cations (FA^+^ and MA^+^) and their influence on crystal structure and stability, as well as the effect of spin speed during the second step on film morphology.^18,19^ In inverted device architectures, lowering the annealing temperature is particularly important, as the self-assembled monolayers (SAMs) commonly employed as hole transport layers tend to detach under high-temperature treatment. Very recently, strategies have been proposed to facilitate low-temperature crystallization, such as introducing ionic-liquid additives into the PbI_2_ precursor to form reactive porous coordination complexes.^20^ In addition, passivation approaches employing organic ammonium bromide additive or bulky organic ammonium iodide additive at the hole-transport layer interface have been shown to suppress interfacial defects and improve optoelectronic properties, thereby contributing to enhanced device efficiency.^21^ Despite these advances, the influence of precursor solution temperature during the casting step of the two-step spin-coating process on crystallization behavior, film quality, and overall device performance remains insufficiently explored.

In this work, we focus on elucidating the previously overlooked yet critical role of precursor solution temperature in the two-step spin-coating process for perovskite solar cells. By systematically varying the temperatures of both PbI_2_ and FAI precursor solutions, we investigate their effects on crystallization behavior, film morphology, phase composition, and photovoltaic performance. Combining microscopic, spectroscopic, and *in situ* photoluminescence analyses, we reveal the temperature-dependent mechanisms governing perovskite film formation and device operation. These findings provide new insights into the processing–structure–property relationship and establish practical guidelines for producing high-quality FAPbI_3_ thin films through precise temperature control during spin coating.

## 2 Results and discussion

### 2.1. Overview of perovskite film fabrication

**Fig. 1:**
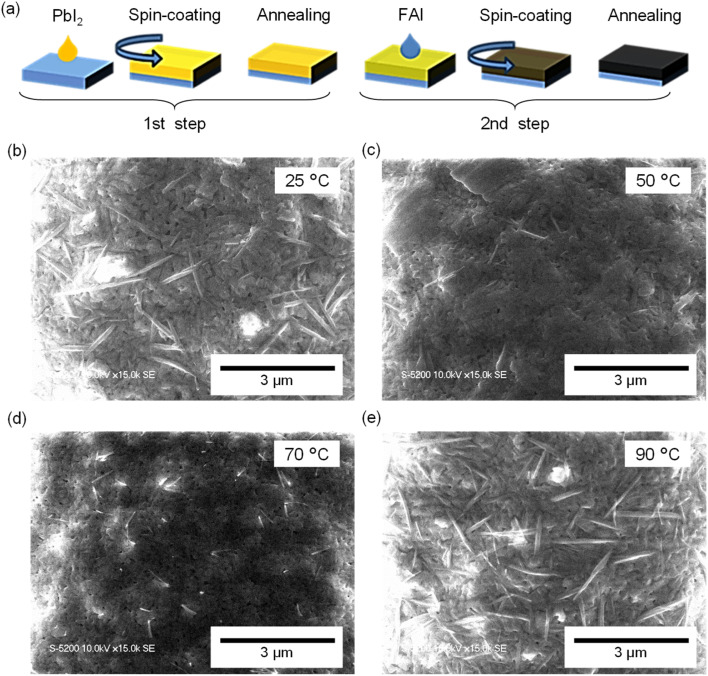
(a) Schematic illustration of the two-step procedure for fabricating FAPbI_3_ films. (b–e) SEM images of PbI_2_ surfaces prepared from precursor solutions at different temperatures: 25 °C (b), 50 °C (c), 70 °C (d), and 90 °C (e).

FAPbI_3_ perovskite thin films were fabricated using a two-step spin-coating method, as illustrated in Fig. 1(a). In this process, a solution of the inorganic precursor PbI_2_ was first spin-coated onto a pre-cleaned glass substrate and thermally annealed to remove residual solvent. Subsequently, the organic precursor FAI was spin-coated onto the PbI_2_ layer, followed by a final annealing step under ambient conditions to complete perovskite film formation. Detailed fabrication procedures are provided in Note S1 in the SI.

The PbI_2_ precursor solution was doped with 5 mol% RbCl, resulting in the formation of the electronically inactive (PbI_2_)_2_RbCl by-product. The presence of this phase at grain boundaries and surfaces has been reported to suppress charge recombination, mitigate current–voltage hysteresis, and enhance the structural and environmental stability of the perovskite phase.^22^ To further promote the growth of the stable α-phase of FAPbI_3_, methylammonium chloride (MACl) was added to the organic precursor solution at a concentration of 18 mg mL^−1^. MACl is known to form transient intermediate phases that facilitate the perovskite conversion process, thereby enabling reproducible fabrication of the desired α-FAPbI_3_ structure.^23–26^

### 2.2. Effect of PbI_2_ precursor solution temperature

We varied the temperature of the PbI_2_ precursor solution during the first-step spin-coating, *T*_1_, to 25, 50, 70, and 90 °C. The surface morphology of the resulting PbI_2_ films was examined using scanning electron microscopy (SEM), as shown in Fig. 1(b)–(e). Although all films exhibited a porous structure, distinct temperature-dependent differences were observed. Needle-like crystals with lengths of approximately 1.5 µm appeared at *T*_1_ = 25 and 90 °C, whereas such features were scarcely observed in films deposited at *T*_1_ = 50 and 70 °C. These results demonstrate that the precursor solution temperature exerts a pronounced influence on PbI_2_ film morphology. At relatively low *T*_1_ (25 °C), the limited solubility of PbI_2_ leads to rapid precipitation, resulting in the formation of highly crystallized needle-like structures. At *T*_1_ = 90 °C, the accelerated solvent evaporation induces rapid nucleation and crystal growth, again yielding needle-like morphologies.

**Fig. 2:**
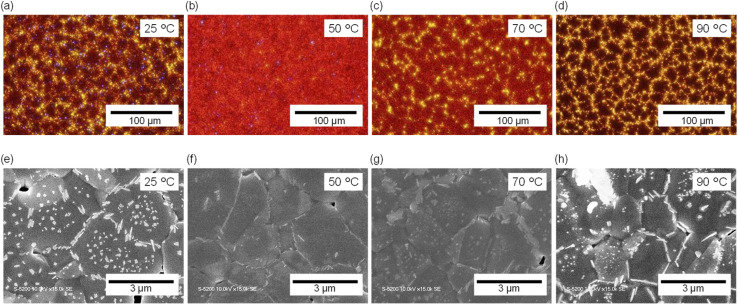
(a–d) Optical microscopy images of FAPbI_3_ surfaces. (e–h) SEM images of FAPbI_3_ surfaces. The PbI_2_ precursor solution temperatures used to fabricate the underlayer were 25 °C (a and e), 50 °C (b and f), 70 °C (c and g), and 90 °C (d and h).

Subsequently, in the second step, the FAI precursor solution (maintained at 25 °C) was spin-coated onto each PbI_2_ film to form perovskite films. The surface morphology of the resulting films was examined using optical microscopy [Fig. 2(a)–(d)]. As described in Fig. S1 in the SI, the pinhole density is decreased with increased solution temperature, whereas the areal fraction of yellow regions—corresponding to unreacted PbI_2_ or thinner perovskite areas—is as high as 3–5% for films prepared at *T*_1_ = 25, 70, and 90 °C, but only ∼0.05% for the film at *T*_1_ = 50 °C. Overall, the film fabricated at *T*_1_ = 50 °C exhibited the best quality, characterized by a nearly pinhole-free and highly uniform morphology. At *T*_1_ = 70 °C, the surface was relatively uniform but still contained residual unreacted PbI_2_ domains, while the conditions of *T*_1_ = 25 and 90 °C result in poor-quality films. These results clearly demonstrate that the PbI_2_ precursor solution temperature during the first deposition step directly governs the quality of the subsequently formed perovskite thin films.

In addition, scanning electron microscopy (SEM) was performed to gain microscopic insight into the surface morphology [Fig. 2(e)–(h)]. The observed needle-like nanocrystals correspond to residual PbI_2_.^22^ The density of residual PbI_2_ nanocrystals was estimated to be ∼5.86 µm^−2^ at *T*_1_ = 25 °C, ∼1.42 µm^−2^ at 50 °C, ∼4.83 µm^−2^ at 70 °C, and ∼7.33 µm^−2^ at 90 °C (Fig. S2 in the SI), which is consistent with the values estimated from optical microscopic image. These results indicate that precursor-solution temperature exerts strongly influence on the spatial distribution of PbI_2_ and its subsequent reactivity with the organic precursor. In particular, under the conditions of *T*_1_ = 25 and 90 °C, SEM images of the first-step PbI_2_ films [see Fig. 1(b) and (e)] revealed large needle-like crystals, which likely hinder complete reaction with FAI during the second step, leaving unreacted PbI_2_ residues. In contrast, at *T*_1_ = 50 and 70 °C, the PbI_2_ layers formed in the first step exhibited denser and more uniform morphologies, enabling more homogeneous interdiffusion and reactions. As a result, the density of residual PbI_2_ was markedly reduced. Notably, the lowest density of residual PbI_2_ was achieved at *T*_1_ = 50 °C, indicating an optimal balance between controlled crystal growth and high reactivity.

**Fig. 3:**
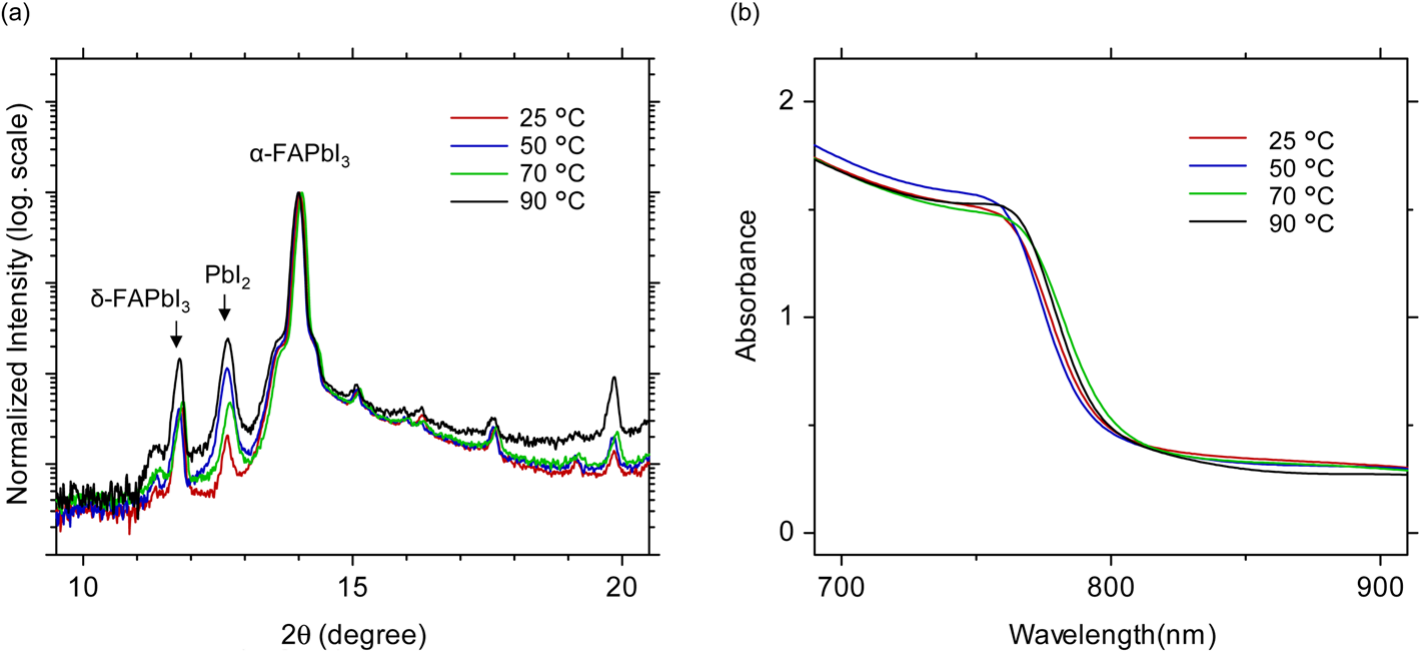
Structural and spectroscopic characterizations of FAPbI_3_ films fabricated by the two-step process at different PbI_2_ precursor solution temperatures in the first step. (a) XRD patterns. The vertical axis is on a logarithmic scale. (b) Absorbance spectra around FAPbI_3_ band edge.

To further evaluate the crystal structure and secondary phases, X-ray diffraction (XRD) measurements were performed [Fig. 3(a)]. The patterns are plotted on a logarithmic intensity scale and normalized to their maximum values for comparison. The diffraction peak at 14.0° corresponds to the (001) plane of the α-FAPbI_3_ phase, while peaks at 11.7° and 12.6° are assigned to the δ-FAPbI_3_ and PbI_2_ phases, respectively. At *T*_1_ = 25 °C, the δ-FAPbI_3_ peak exhibits higher intensity than that of PbI_2_, indicating dominant δ-phase formation accompanies by insufficient crystallization of the α-phase perovskite. At *T*_1_ = 50 °C, the δ-FAPbI_3_ signal intensity is similar to that at 25 °C, although unreacted PbI_2_ remains. At *T*_1_ = 70 °C, the 11.7° and 12.6° peaks appear with comparable intensities, suggesting a competitive coexistence of δ-FAPbI_3_ and PbI_2_. At *T*_1_ = 90 °C, both impurity peaks are most pronounced, reflecting substantial phase inhomogeneity and incomplete phase conversion. These results demonstrate that the precursor solution temperature in the first spin-coating step critically governs the formation of the α-FAPbI_3_ phase and the persistence of impurity phases.

Absorbance measurements reveal that FAPbI_3_ films exhibit absorption edge around 800 nm [Fig. 3(b)]. No significant differences were observed as a function of the PbI_2_ precursor-solution temperature. Nevertheless, the film prepared at *T*_1_ = 50 °C exhibits a relatively higher overall absorbance, consistent with the optical microscopic observation [Fig. 2(b)]. The elevated baseline observed at wavelengths above 800 nm is due to optical scattering and suppressed at higher solution temperatures, correlating with the reduced pinhole density.

**Fig. 4:**
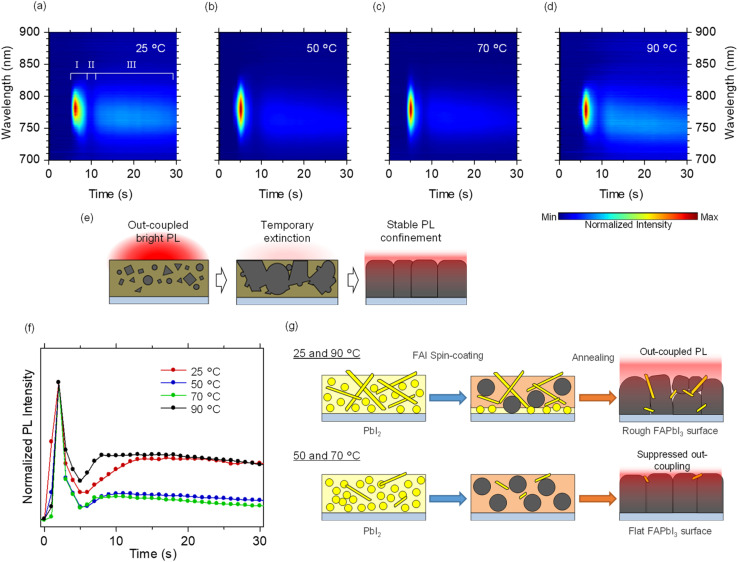
(a–d) Colormaps of *in situ* PL spectra recorded during the annealing process in the second step. The PbI_2_ precursor solution temperatures in the first step were 25 °C (a), 50 °C (b), 70 °C (c), and 90 °C (d). The Roman numerals denoted in (a) denote three stages within perovskite crystallization process. (e) Schematic illustration of perovskite crystal growth and PL outcoupling during the second step annealing process. (f) Temporal evolution of *in situ* PL peak intensity, normalized to the maximum for each dataset. (g) Schematics of perovskite film formation models in the second step. The upper and lower panels correspond to growth models for first-step solution temperatures of 25 or 90 °C, and 50 or 70 °C, respectively.

To analyze the crystallization process from the PbI_2_ underlayer to the perovskite film in real time, we monitored the temporal evolution of the photoluminescence (PL) spectrum during annealing in the second step (after FAI deposition) [Fig. 4(a)–(d)]. The excitation source for the *in situ* PL measurements was a continuous-wave laser with a wavelength of 405 nm. Under all temperature conditions, three characteristic stages of PL evolution were consistently observed, each corresponding to a distinct phase in the perovskite crystallization process [Fig. 4(e)].

In the first stage [see Fig. 4(a)], strong PL emission appeared at the early stage of the reaction, attributed to radiative recombination within high-density amorphous or quasi-crystalline perovskite nuclei. In the second stage, the PL intensity gradually decreased, which is primarily attributed to two factors:^27,28^ (i) solvent evaporation during annealing can lead to partial re-dissolution of the seed crystals, reducing their density; and (ii) Ostwald ripening incorporates smaller nuclei into larger crystals, thereby lowering the overall nucleation density.^27,29^ In the third stage, PL emission re-emerged as solvent evaporation was completed and the phase transition to α-FAPbI_3_ was achieved. However, the PL intensity at this stage was weaker than in the initial stage, due to reduced light-outcoupling efficiency as a dense and optically flat perovskite layer formed.^30^

Fig. 4(f) highlights the distinct differences in the temporal PL evolution at different precursor solution temperatures. Under the conditions of *T*_1_ = 50 and 70 °C, the initial PL emission was strong, whereas the steady-state emission at later times was markedly weakened. This behavior indicates that the FAI precursor solution penetrated more deeply into the PbI_2_ layer, facilitating a more complete and efficient conversion reaction. The resulting higher nucleus density likely gives rise to the strong initial PL signal. The decrease in PL intensity in the second stage was also pronounced at *T*_1_ = 50 and 70 °C; the emission decay constants were below 1.5 s, while the emission decay at *T*_1_ = 25 and 90 °C were more slowly (>∼2.0 s, see Fig. S3 in the SI). In the third stage, the formation of a dense, continuous, and optically smooth film was promoted at *T*_1_ = 50 and 70 °C, leading to enhanced optical confinement and reduced light extraction. In contrast, under the conditions of *T*_1_ = 25 and 90 °C, light was more readily extracted from the film, consistent with a rougher surface morphology [see Fig. 4(g)]. Fig. S4 in the SI exhibits a temporal evolutions of PL peak positions. Fluctuations in the peak position at the third stage were pronounced at *T*_1_ = 25 and 90 °C, whereas at *T*_1_ = 50 and 70 °C the peak position remains stable at 760 nm, demonstrating that composition fluctuations during annealing are suppressed well.

Fig. S5 in the SI is an example demonstrating the effect of other solution preparation condition on thin film formation. We investigated an influence of concentration of MACl added into the precursor solution. MACl addition has a pronounced impact on the *in situ* PL, indicating a strong influence on perovskite formation dynamics and the development of optically smooth, mirror-like films. This result confirms that the *in situ* PL is a convenient and powerful tool for evaluating the crystallization and film formation dynamics.

In addition to morphological and structural evaluations described above, we conducted a proof-of-concept study on photovoltaic characteristics (see Note S2 and Fig. S6 in the SI). A solar cell device prepared at *T*_1_ = 70 °C exhibited the most favorable performance with minimal reproducibility. Taken together, these results clearly demonstrate that precursor solution temperature is a critical processing parameter that directly governs film quality, defect density, and the photovoltaic performance of FAPbI_3_-based perovskite solar cells.

### 2.3. Effect of FAI precursor solution temperature

Next, we evaluated the reactivity in the second step of perovskite film formation. The temperature of the FAI precursor solution at the second step, *T*_2_, varied to 25, 50, 70, and 90 °C, while *T*_1_ was fixed at 70 °C. Perovskite films were then fabricated under each *T*_2_ condition for comparative analysis.

**Fig. 5:**
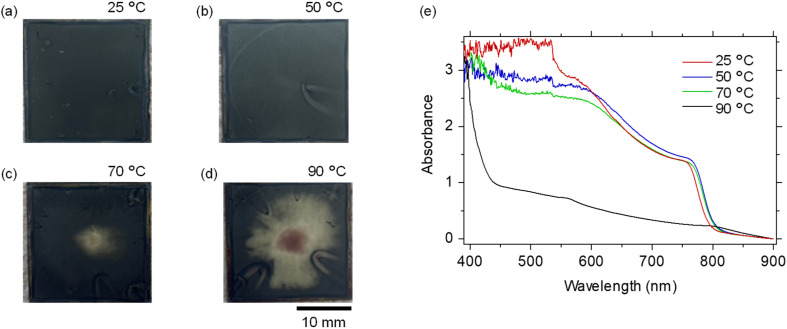
(a–d) Images of perovskite films prepared with different FAI precursor solution temperatures: 25 °C (a), 50 °C (b), 70 °C (c), and 90 °C (d). (e) UV-vis absorbance spectra of perovskite films as a function of the second-step solution temperature.

Fig. 5(a)–(d) show images of the perovskite films when *T*_2_ was scanned. At *T*_2_ = 25 °C, the entire film appeared black and uniformly coated, indicating successful perovskite formation. At *T*_2_ = 50 °C, slight transparency was observed at the center of the film, although the overall appearance remained uniform. At *T*_2_ = 70 °C, the central transparent region became more pronounced, while at *T*_2_ = 90 °C the film exhibited a reddish discoloration at the center surrounded by a transparent area. These distinct color changes suggest variations in phase composition and reaction uniformity during perovskite formation. Microscopic morphological evaluations were also carried out as shown in Fig. S7 in the SI. Optical microscopic and SEM analyses revealed that the pinhole density increased progressively with increasing FAI-precursor solution temperature. At *T*_2_ = 90 °C, finally, pronounced surface degradation was observed, indicative of a temperature-induced phase transition in the perovskite structure.

To evaluate the optical properties and phase composition of the films, UV-vis absorption measurements were conducted [Fig. 5(e)]. The films prepared at *T*_2_ = 25–70 °C exhibited the absorption edge of α-FAPbI_3_ around 800 nm. In contrast, at *T*_2_ = 90 °C, the characteristic absorption peak around 800 nm almost completely vanished, while an absorption edge emerged near 440 nm. This feature indicates that the reddish discoloration observed in the optical microscopy image of the film prepared at *T*_2_ = 90 °C originated from the formation of the non-perovskite δ-FAPbI_3_ rather than the photoactive α-FAPbI_3_. Under the high-temperature condition, the reaction proceeded too rapidly during spin coating, favoring δ-phase stabilization before the α-phase transition could be completed during subsequent annealing.

Furthermore, the absorption peak located around 540 nm showed a gradual decrease in intensity with increasing *T*_2_. This observation suggests that elevating the solution temperature in the second step effectively accelerates the residual PbI_2_ content within the perovskite films. These results demonstrate that the precursor solution temperature in the second step also strongly influences the reaction kinetics between PbI_2_ and FAI during spin coating, thereby significantly affecting both the type of perovskite phase formed and the resulting crystal quality.

## 3 Conclusions

In conclusion, we systematically clarified the decisive role of precursor solution temperature in the two-step spin-coating fabrication of FAPbI_3_ perovskite thin films. By varying the PbI_2_ precursor solution temperature between 25 and 90 °C, we found that intermediate temperatures of 50–70 °C yield compact PbI_2_ underlayers, leading to a marked reduction in residual PbI_2_ nanocrystals from ∼5–7 µm^−2^ (25 and 90 °C) to ∼1.4 µm^−2^ at 50 °C. Correspondingly, the areal fraction of unreacted or thin regions in the final perovskite films was minimized to ∼0.05% at 50 °C, compared with 3–5% under non-optimal conditions. *In situ* photoluminescence measurements revealed three distinct crystallization stages for all conditions, with pronounced temperature-dependent differences in PL intensity evolution. Films prepared from PbI_2_ solutions at 50–70 °C exhibited strong initial PL emission followed by significant suppression at later stages, consistent with dense film formation and reduced optical out-coupling.

Furthermore, varying the FAI precursor solution temperature from 25 to 90 °C demonstrated that low-temperature FAI solutions (25 °C) favor stabilization of the photoactive α-FAPbI_3_ phase, while higher temperatures induce increased pinhole density and δ-phase formation, as evidenced by the disappearance of the ∼800 nm absorption edge at 90 °C. Although device efficiencies were not fully optimized, reproducible temperature-dependent trends were observed, with the best-performing devices obtained for PbI_2_ and FAI solution temperatures of 70 °C and 25 °C, respectively.

These results establish precursor solution temperature as a simple yet powerful processing parameter for controlling crystallization dynamics, morphology, and phase purity in two-step deposited FAPbI_3_ films, providing practical guidelines for reproducible perovskite thin-film fabrication.

## Conflicts of interest

The authors declare no conflict of interests.

## Supplementary Material

RA-016-D5RA08868B-s001

## Data Availability

The data for this manuscript will be deposited in the institutional repository of Kyoto Institute of Technology, which will become available soon. Supplementary information (SI) is available. See DOI: https://doi.org/10.1039/d5ra08868b.
