# Supplementary material for: Impact of precursor solution temperature on two-step spin-coated FAPbI3 film elucidated by surface morphology and in situ photoluminescence dynamics
Source: RSC Adv. 2026 Jan 22;16(6):4841–7. doi: 10.1039/d5ra08868b (PMC12825113; doi:10.1039/d5ra08868b)
Supplement: RA-016-D5RA08868B-s001 [file RA-016-D5RA08868B-s001.pdf]

Supplementary Information (SI) for RSC Advances.
This journal is © The Royal Society of Chemistry 2026

Supplementary Materials

Impact of precursor solution temperature on two-step spin-coated $FAPbI_3$ film elucidated by surface morphology and in-situ photoluminescence dynamics

R. Okuyama, K. Uezono, Y. Inada, T. Yamao, D. Okada, and K. Yamashita

Contents

Supplementary Note S1 Materials and methods.

Figure S1 Optical microscopic observations and analyses.

Figure S2 Scanning electron microscopic observations and analyses.

Figure S3 PL decay during annealing process.

Figure S4 PL peak positions during annealing process.

Figure S5 Impact of MACl additive on in-situ PL dynamics.

Supplementary Note S2 Photovoltaic characterizations.

Figure S6 Current density – voltage measurements.

Figure S7 Impact of second-step solution temperature on surface morphology.

Supplementary Note S1

Materials

SnO_2 colloid precursor [tin (IV) oxide, 15% in H_2O colloidal dispersion] was purchased from Alfa Aesar. Formamidinium iodide (FAI), methylammonium chloride (MACl), phenethylammonium iodide (PEAI), lead (II) Iodide (PbI_2), rubidium chloride (RbCl) and tris[2-(1H-pyrazol-1-yl)-4-tert-butylpyridine]cobalt(III) tris[bis(trifluoromethylsulfonyl)imide] (FK209) were purchased from TCI. 2,2′,7,7′-tetrakis[*N*,*N*-di(4-methoxyphenyl)amino]-9,9′-spirobifluorene (spiro-OMeTAD), bis(trifluoromethane)sulfonimide lithium salt (Li-TFSI) and 4-tertbutylpyridine (tBP) were purchased from Sigma Aldrich. Except for SnO_2 solution stored in ambient air, all the materials are stored in the nitrogen filled glove box to avoid the water.

Perovskite film deposition and device fabrication

A glass slide coated with indium tin oxide (ITO) film was used as the substrate and sequentially cleaned by ultrasonic washing with detergent, deionized water, acetone, and 2-propanol (IPA), followed by ultraviolet-ozone treatment for 15 min. Then the substrate was spin-coated with a thin layer of SnO_2 nanoparticle (7.5 % diluted by water) at 4,000 rpm for 30 s. Thermal treatment at 150 °C for 30 min was performed in ambient air.

As the first step in the two-step deposition method, a solution of PbI_2 with a concentration of 1.5 M was spin-coated onto SnO_2 film at 1,500 rpm for 30 s, followed by thermal treatment at 70 °C for 1 min, in a nitrogen-filled glove box. We used a mixed solvent of *N,N*-dimethylformamide and dimethyl sulfoxide (DMF and DMSO) with a mixing ratio of 9:1. RbCl was added into the PbI_2 solution with a concentration of 5 mol%. Here the solution temperature was controlled by a hotplate with temperature control function and varied in a range of 25–90 °C.

For $FAPbI_3$ perovskite film formation in the second step, a solution of FAI:MACl (90 mg:18 mg in 1ml of IPA) was spin-coated onto the PbI_2 layer at 1,800 rpm for 30 s. After the spin coating, the sample was taken out from the nitrogen-filled glove box to atmospheric environment for thermal annealing at 150 °C for 15 min in humidity conditions (30–40 %). Then, the sample was

transferred again to the glove box for further processing. To form a passivation layer, the PEAI solution was dissolved in IPA with 20 mM and spin-coated onto the perovskite surface at a spin rate of 5,000 rpm for 30 s without any further processing.

Then the hole transporting layer was deposited on top of the passivation layer using a solution of spiro-OMeTAD at 1,500 rpm for 30 s. This process was also performed in the glove box. The solution consisted of 90 mg of spiro-OMeTAD, 23 μl of a stock solution of Li-TFSI (520 mg of Li-TFSI in 1 ml of acetonitrile), 10 μl of a stock solution of FK209 (300 mg FK209 in 1ml acetonitrile), 39.5 μl of a chlorobenzene solution of tBP. Finally, Au film was thermally evaporated as a counter electrode using a shadow mask. The device size area was ~0.12 cm^2.

Optical characterization

In-situ photoluminescence (PL) measurements were performed by using a laser light source with a wavelength of 532 nm. The excitation laser light was introduced into a bifurcated fiber bundle with a core diameter of 100 μm. The sample was put on a temperature-controlled hotplate and excited by the excitation light output from the fiber bundle. PL signal was collected with the same fiber bundle and measured by using a spectrometer (USB4000, Ocean Optics).

Device characterization

To evaluate photovoltaic properties, we used a solar simulator (XES-40SI, SAN-EI) and a source meter (Model 2400, Keythley). The illumination condition was 100 $mW\cdot cm^{-2}$ of AM1.5G. The current density-voltage curves measured under both the forward and reverse voltage scanning directions were recorded.

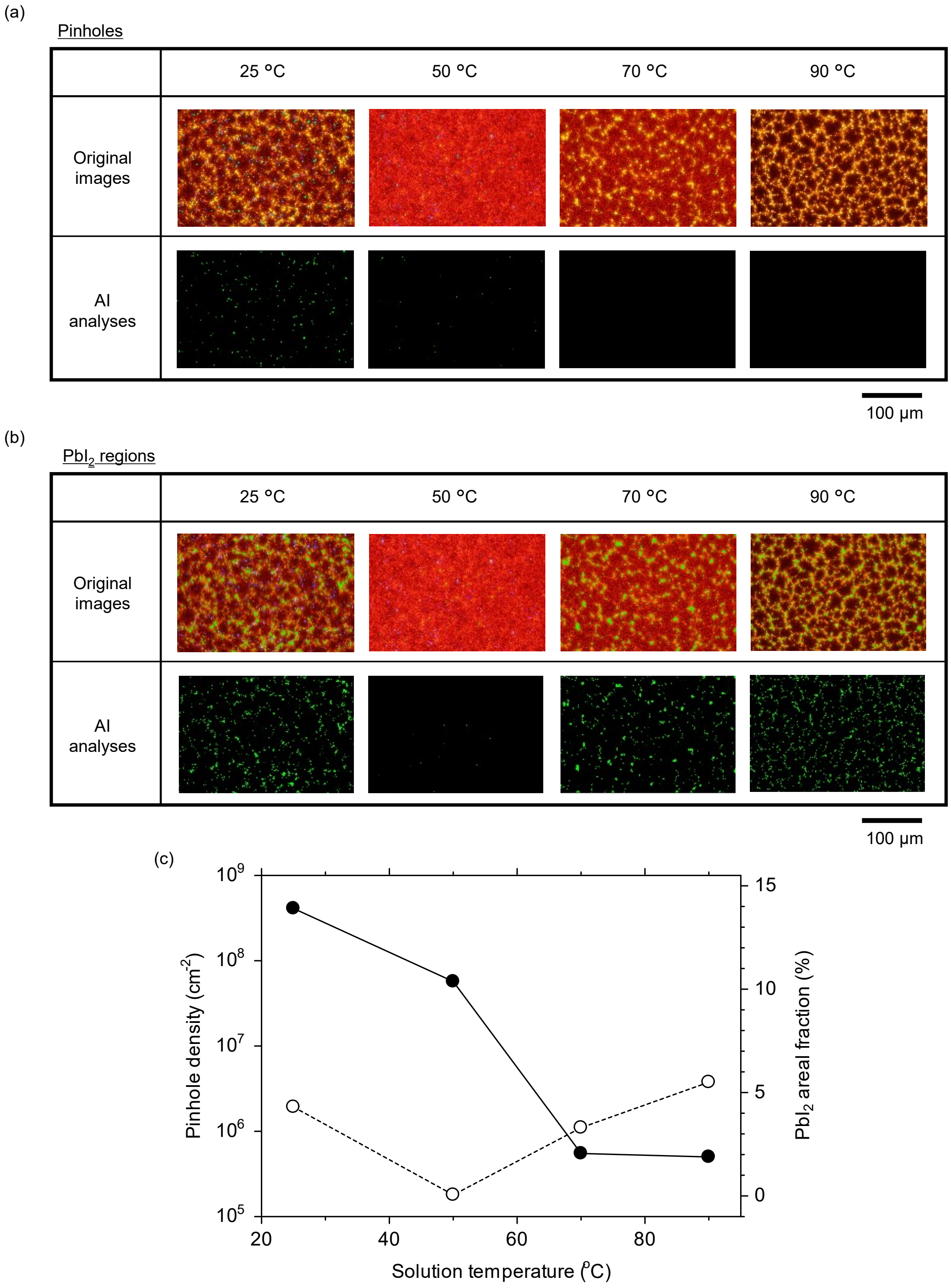

Figure S1 Microscopic image analyses of the surface morphology of perovskite thin films prepared with PbI_2 precursor solutions at various temperatures. (a) Analysis of pinhole

density. The pinholes are observed as pale blue spots within the image. (b) Analysis of surface coverage of PbI_2 residual layer. We identified yellow regions as the PbI_2. These image analyses were performed using AI. (c) Summary of pinhole density (closed circles) and PbI_2 areal fraction (open circles) depending on solution temperature.

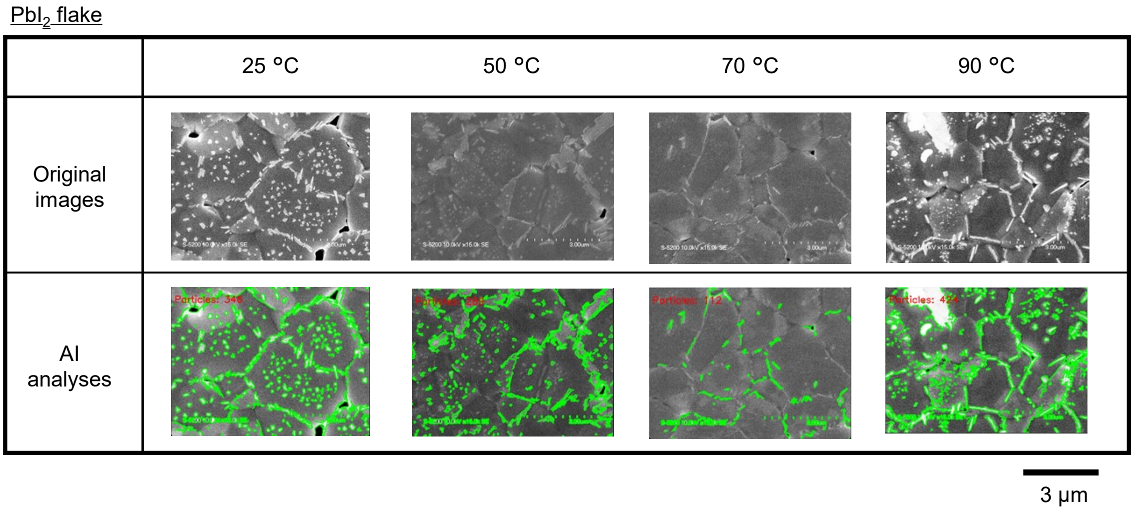

Figure S2 SEM image analyses of perovskite thin films prepared with PbI_2 precursor solutions at various temperatures. We identified residual PbI_2 crystal flakes and evaluated its surface density using AI.

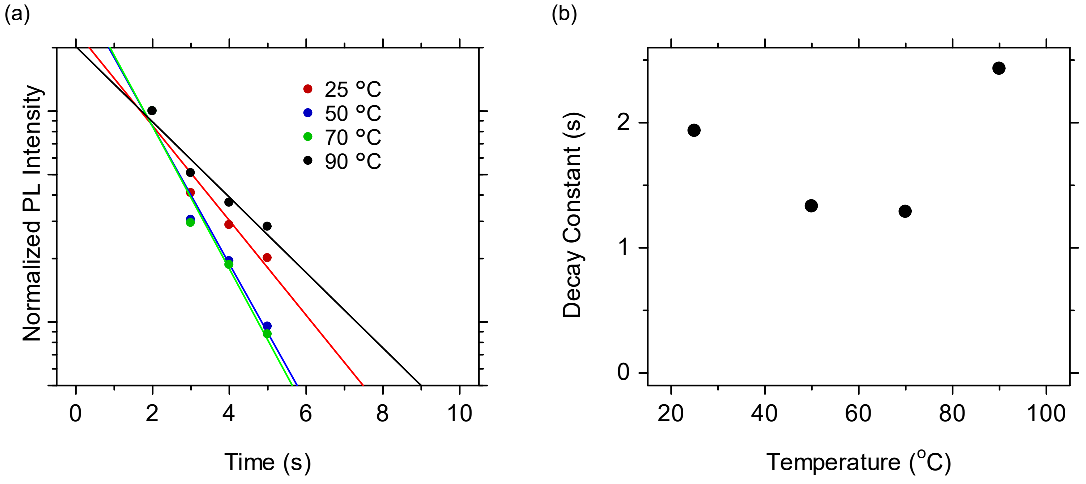

Figure S3 PL decay constant in the second stage during annealing. (a) Logarithmic plots of PL intensity evolution at the different solution temperatures. Solid lines show exponential fitting functions. (b) PL decay constant evaluated by the fitting analyses as a function of solution temperature.

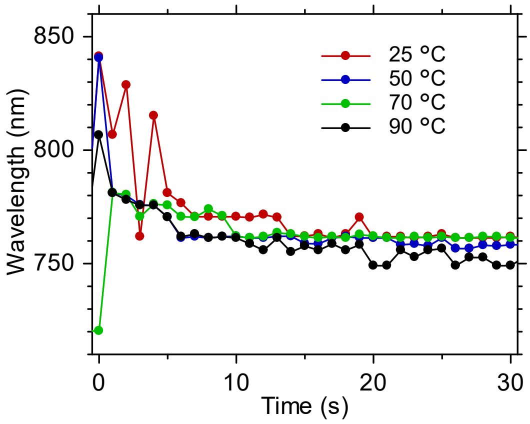

Figure S4 Temporal evolutions of PL peak wavelength during annealing extracted from colormap data shown in Fig. 4 in the main manuscript.

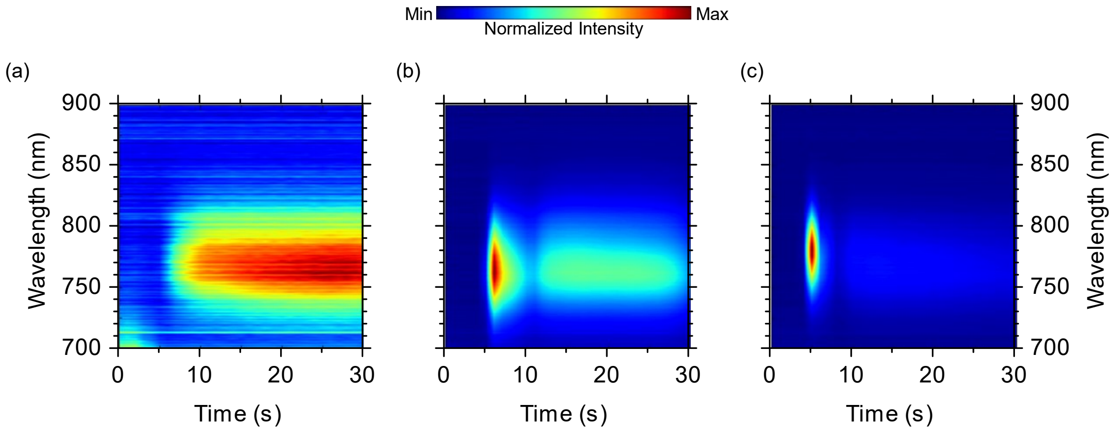

Figure S5 Colormaps of in-situ PL spectra for $FAPbI_3$ perovskite recorded during the annealing process in the second step. (a) A result obtained using a precursor solution without MACl. (b and c) Results when using solutions with MACl addition amounts of 9 ml (b) and 18 ml (c).

Supplementary Note S2

Current density–voltage (*J*–*V*) characteristics were measured for devices with a structure of ITO/SnO_2/perovskite/PEAI/spiro-OMeTAD/Au. As we have not yet optimized the fabrication processes for solar cell devices, the power conversion efficiency (PCE) is not sufficiently high. Nevertheless, as shown in Fig. S6a reveals the solar cell devices exhibit reasonable electrical characteristics without hysteresis. As shown in Fig. S6b, the average PCEs were evaluated as 5.57% at 25 °C, 6.01% at 50 °C, 12.3% at 70 °C, and 10.7% at 90 °C, demonstrating a clear correlation between precursor solution temperature and device performance. A representative device fabricated at 70 °C achieved a short-circuit current density of 20.8 mA·cm^{-2}, an open-circuit voltage of 1.03 V, a fill factor of 0.72, and a PCE of 15.5% (Fig. S6a). Under this optimized condition, controlled precursor temperature effectively suppressed residual PbI_2 while promoting favorable crystal growth and high film uniformity, thereby yielding consistently high PCE values. In contrast, devices fabricated at 50 °C exhibited extremely low residual PbI_2 density, as confirmed by SEM and XRD

analyses. This condition likely resulted in insufficient grain boundary passivation, still promoting pinhole formation (see Fig. S1) and enhancing charge recombination and leakage currents, which in turn reduced the final device efficiency.

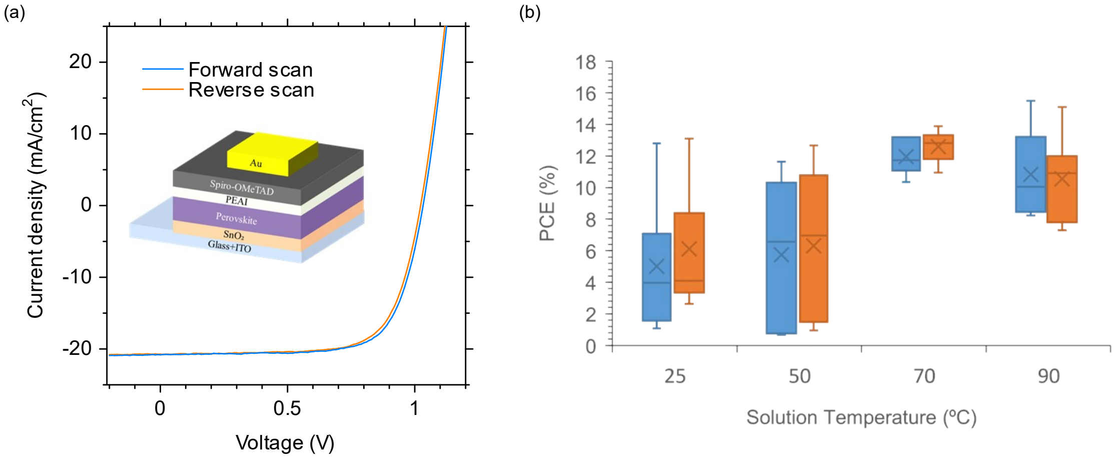

Figure S6 Solar cell device evaluations. (a) Current density – voltage curves of the best sample in this study. Solution temperature was 70 °C. A short-circuit current density of 20.8 mA·cm^{-2}, an open-circuit voltage of 1.03 V, a fill factor of 0.72, and a PCE of 15.5% were obtained. (b) Boxplots of PCEs varied with the solution temperatures. Blue and orange plots show the data of forward-scan and reverse-scan measurements, respectively.

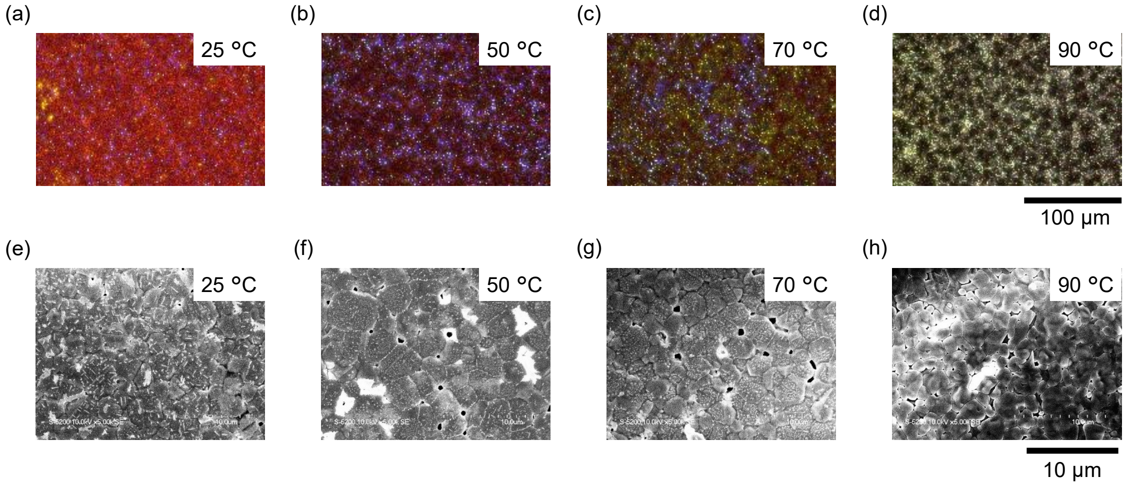

Figure S7 Microscopic morphological evaluations of $FAPbI_3$ surfaces. (a–d) Optical microscopy images. (e–h) SEM images. The FAI precursor solution temperatures used to fabricate the perovskite layers were 25 °C (a, e), 50 °C (b, f), 70 °C (c, g), and 90 °C (d, h).
